# HMGA2 sustains self-renewal and invasiveness of glioma-initiating cells

**DOI:** 10.18632/oncotarget.9744

**Published:** 2016-05-31

**Authors:** Xiaoling Zhong, Xuan Liu, Yamu Li, Man Cheng, Wen Wang, Kuan Tian, Lili Mu, Tao Zeng, Ying Liu, Xiaobing Jiang, Luyang Yu, Liang Gao, Yan Zhou

**Affiliations:** ^1^ Hubei Key Laboratory of Cell Homeostasis, College of Life Sciences at Wuhan University, Wuhan 430072, China; ^2^ Department of Neurosurgery, The Tenth Affiliated Hospital, Tongji University, Shanghai 200072, China; ^3^ Department of Neurosurgery, Union Hospital, Tongji Medical College, Huazhong University of Science and Technology, Wuhan 430022, China; ^4^ College of Life Sciences, Zhejiang University, Hangzhou, Zhejiang 310058, China

**Keywords:** glioma, cancer stem cell, pericyte, self-renewal, cell invasion

## Abstract

Glioblastoma multiforme (GBM) is the most common type of brain tumors with dismal outcomes. The mesenchymal phenotype is the hallmark of tumor aggressiveness in GBMs. Perivascular smooth muscle cells (pericytes) are essential in homeostasis of normal and glioma tissues. Here we found HMGA2, an architectural transcription factor that promotes mesenchymal phenotypes in a number of solid tumors, is highly expressed in mesenchymal subtype of GBMs and labels both glioma pericytes and glioma-initiating cells (GICs). Accordingly, depletion of *HMGA2* in GICs resulted in compromised self-renewal and tumorigenic capability, as well as undermined mesenchymal or pericyte differentiation. We further showed HMGA2 allows expressions of *FOXM1* and *PLAU* to maintain GIC propagation, gliomagenesis and aggressiveness both *in vitro* and *in vivo*. Therefore, suppressing HMGA2-mediated GIC self-renewal and invasiveness might be a promising means to treat GBMs.

## INTRODUCTION

Gliomas make up about 30% of all brain and central nervous system tumors and 80% of primary malignant brain tumors, and they are essentially incurable [1]. Glioblastoma multiforme (GBM), the most common type of glioma, is highly aggressive and associated with very poor survival outcomes [2, 3]. Local invasiveness and neo-angiogenesis are hallmarks of aggressiveness of GBMs [4, 5]. Mesenchymal features including uncontrolled ability to invade and stimulate angiogenesis, and the corresponding molecular signatures such as alterations of *Neurofibromatosis-1* (*NF1*) expression can be found in a portion of GBMs [6]. These mesenchymal (MES) GBMs are mostly primary tumors and, in some studies, display a worse prognosis than proneural (PN) GBMs, which have higher expression of *PDGFR* along with *IDH1* point mutations [7, 8]. A subset of radio- and chemo-resistant cells have been characterized in the GBM bulk, which exhibit stem-like features such as stem cell biomarker expression, self-renewal, differentiation upon mitogen retraction and intracranial GBM formation in xenografted immunocompromised mice [9–11]. Interestingly, these so-called glioma-initiating cells or glioma stem cells (GICs/GSCs) isolated from MES or PN GBMs usually generate xenograft tumors with MES or PN features respectively [12]. Recent studies revealed that mesenchymal phenotypes of GICs could be induced by master transcription factors (TFs) including Signal transducer and activator of transcription 3 (STAT3), CCAAT enhancer-binding protein-β (C/EBPβ), and Transcriptional coactivator with PDZ-binding motif (TAZ) [13, 14]. In addition, the expressions of these master TFs were induced in GICs by TNF-α secreted by infiltrating macrophages/microglia to promote mesenchymal differentiation and radiation resistance [15].

Similar to features of neural progenitor/stem cells in embryonic and adult brain, GICs preferentially resides in close proximity to tumor microvasculature, which could provide favorable environment (niche) [16]. Most normal and tumor microvessels have two distinct but interdependent cellular components, endothelial cells (ECs) and contractile perivascular mural cells called pericytes. The crosstalk between ECs and pericytes via direct physical contact and paracrine signaling helps to maintain vessel structures and functions [17]. However, the tumor microvessels often exhibit structural and functional anomalies with irregular pericytes on endothelial tubules or microvasculature consisting of pericytes only but lacking ECs [18]. Moreover, the GICs are capable of generating ECs and pericytes both *in vivo* and *in vitro*, further supporting the cellular hierarchies in GBMs with tumorigenic GICs at the tip [18–21]. Thus, dissecting molecular mechanisms underlying GIC differentiating into mesenchymal and pericyte progenies would be crucial to developing therapeutic means that specifically target invasiveness and neo-angiogenesis.

*HMGA2*, a member of the high-mobility group A (HMGA) family, encodes a small, chromatin-associated protein that preferentially binds to AT-rich stretches of B-form DNA via its “AT hooks”. HMGA2 can modulate transcription by altering chromatin structure and through protein-protein interactions [22–24]. HMGA2 plays crucial roles in a variety of developmental and tumorigenic processes. *Hmga2* knockout mice exhibit a pygmy phenotype [25]. Knockdown of *Hmga2* and *Hmga1*, a HMGA2 paralogue, in cortical neural progenitor cells (NPCs) results in chromatin condensation and precocious astrocytic differentiation [24]. HMGA2 is also highly expressed in a variety of benign and malignant tumors of mesenchymal and epithelial origin including astrocytomas [23, 26, 27].

Here we explored roles of HMGA2 in maintaining key properties of GICs. Down-regulation of HMGA2 in GICs led to diminished self-renewal and tumorigenic capabilities, as well as undermined mesenchymal or pericyte differentiation. We also characterized novel HMGA2 target genes that may be involved in these processes.

## RESULTS

### HMGA2 is overrepresented in mesenchymal glioblastomas and labels GICs and pericytes

**Figure 1:**
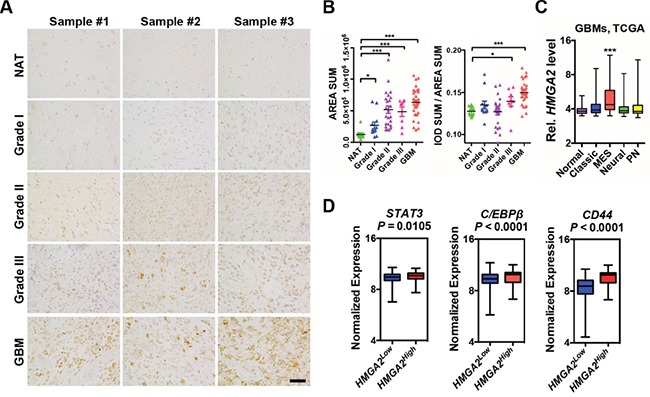
Elevated HMGA2 expression in gliomas **A.** Representative immuno-histochemistry images of HMGA2 expressions in gliomas and normal adjacent brain tissues (NAT) using a tissue array. **B.** Scattered dot plots of total HMGA2+ expression area (left) and intensity (right) of HMGA2 expression in each section. Each sample has two duplicate sections. Measuring and quantifications of IHC images were performed using the Image-pro Plus 6.0 software (Media Cybernetics). **C.** Box and whisker plots showing expressions of *HMGA2* in normal and four subtypes of GBM (grade IV) specimens using data retrieved from TCGA. **D.** Box plots showing normalized expressions of *STAT3*, *C/EBPβ* and *CD44* metagene in *HMGA2*^Low^ and *HMGA2*^High^ GBMs using data retrieved from TCGA. *HMGA2*^low^ (n = 471) and *HMGA2*^high^ (n = 87) expression groups were defined using the minimum P-value approach (maximal chi square statistics approach). P values were determined using a nonparametric Wilcoxon test. IOD, integrated optical density; MES, mesenchymal; PN, proneural. Scale bar: 50 μm.

HMGA2 is highly expressed in a variety of embryonic cells such as in neural progenitor cells but becomes silent in most adult tissues [25, 28]. Consistently, prominent HMGA2 expression can be detected in early (E10.5 – E12.5) embryonic cortical neural precursor cells, but was diminished in mid-gestation (E16.5) mouse cerebrum, subventricular zone (SVZ) and dentate gyrus of adult mice (Supplementary Figure S1A-S1B). Gliomas can be originated from mutated neural precursors [29, 30], and targeting self-renewal of GICs prolongs survival in animal models [31]. We therefore checked HMGA2 expressions in various glioma specimens (6 WHO grade I, 12 grade II, 4 grade III, 13 Grade IV/GBMs) and 5 normal brain tissues on a tissue array using immunohistochemistry followed by quantitation. In contrast to neglected HMGA2 expressions in normal brain tissues (NAT), profound HMGA2 expression can be seen in glioma samples, especially in WHO Grade II-IV gliomas (Figure 1A; Figure 1B, the left panel). Notably, both nuclear and cytosolic HMGA2 can be detected in some samples, which indicates the presence of nuclear localization signal (NLS) truncated forms [32]. We further quantitated HMGA2 densities to take into account denser cellularity in gliomas, and detected significant stronger HMGA2 signals in Grade III and Grade IV (GBM) gliomas (Figure 1B, the right panel). Notably, HMGA2 is enriched in highly aggressive regions of GBMs with typical features of pseudopalisading necrosis, where markers for stem-like cells (SOX2, NESTIN, CD133, β-catenin), proliferation (Ki67 and PH3), microvessels (α-SMA) and invasion (CD44, IBA-1) are also highly expressed (Supplementary Figure S1C-S1D). Next, we examined *HMGA2* expression in four subtypes of GBMs using expression data retrieved from The Cancer Genome Atlas (TCGA). Consistent with HMGA2 roles in mediating EMT in a number of solid tumors, HMGA2 expression is significantly higher only in mesenchymal (MES) GBMs (Figure 1C). Moreover, *HMGA2* expression is positively correlated with expressions of *STAT3* and *C/EBPβ*, two essential transcription factors that promote mesenchymal phenotypes in GBMs; as well as that of *CD44* [33], another hallmark of glioma invasiveness (Figure 1D). Moreover, high HMGA2 expression levels correlate with shorter survival time in glioma patients using the CGGA (The Chinese Glioma Genome Atlas) dataset [34] (Supplementary Figure S1E), which is consistent with reports showing higher levels of IL-6/HMGA2/SOX2 expression indicated shorter overall survival period in GBM patients [35].

**Figure 2:**
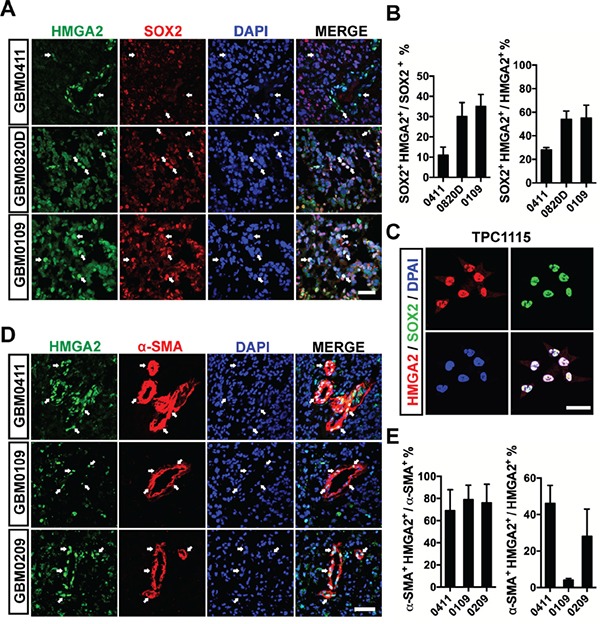
HMGA2 labels GICs and pericytes in glioma tissues **A.** Representative immunofluorescent images showing co-expression of HMGA2 and SOX2 in three GBM samples. **B.** Quantification of co-localization of HMGA2+ and SOX2+ glioma cells of three fields under a 20× object (44,100 μm^2^ per field). **C.** Co-localization of HMGA2 and SOX2 in primary glioma-initiating cells, TPC1115. **D.** Representative immunofluorescent images showing co-expression of HMGA2 and α-SMA in three GBM samples. **E.** Quantification of co-localization of HMGA2+ and α-SMA+ glioma cells of three fields under a 20× object (44,100 μm^2^ per field). Arrows indicate HMGA2-expressing GBM cells. Scale bars: (A and D) 50 μm; (C) 40 μm.

Next, we use immunofluorescent (IF) stainings to study cell types labeled by HMGA2 in GBM samples. First, a significant portion of HMGA2+ cells (24%-55%) express SOX2, a neural stem cell marker; and 11%-35% SOX2+ cells are HMGA2 positive (Figure 2A-2B). Consistently, HMGA2 is ubiquitously co-expressed with SOX2 in adherent cultured glioma-initiating cells (GICs) (Figure 2C). Intriguingly, in some GBM samples, HMGA2-labelled cells have close proximity to tumor microvessels, or even positive for α-SMA/ACTA2 (smooth muscle α-actin), CD146, CD248 and PDGFRB (platelet-derived growth factor receptor beta), which are pericyte (the smooth muscle cell for microvessels) biomarkers (Figure 2D, Supplementary Figure S2A-S2C). As many as 45% HMGA2+ cells are α-SMA positive, and 70-80% α-SMA+ cells express HMGA2 (Figure 2E). However, HMGA2 largely doesn't label CD34+ or CD31+ endothelial cells (ECs, Supplementary Figure S2D). Since GICs can generate glioma pericytes [21], we postulate HMGA2 might have a role in this process.

### HMGA2 maintains self-renewal and tumorigenicity of glioma-initiating cells

**Figure 3:**
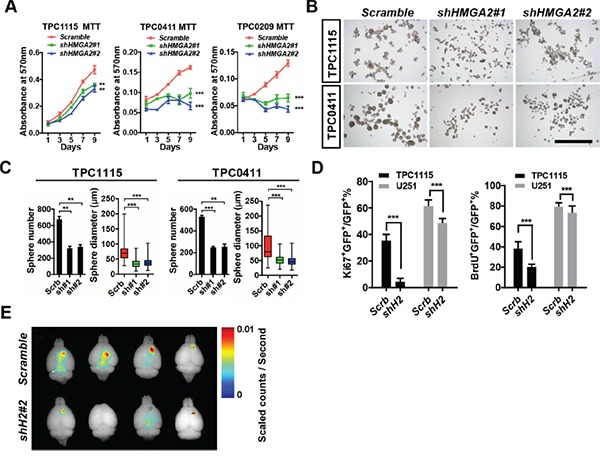
HMGA2 sustains GIC self-renewal and tumorigenicity **A.** MTT assay showing effects of *HMGA2* knockdown on GIC cell propagation in adherent cultures. **B.** Representative images showing TPC1115 and TPC0411 GICs maintained in neurosphere conditions for 7 days after transducing with indicated lentiviruses. **C.** Quantification of sphere numbers and diameters of three independent experiments in (B). **D.** Quantification of Ki67- (left) and BrdU- (right) labeled TPC1115 GICs and U251 glioma cells upon depletion of HMGA2. **E.** Xenografted nude mice were perfused with 4% PFA 10 weeks after intracranial TPC1115 transplantation (1×10^5^) and brains were dissected out. Fluorescent images of brains were captured using the Maestro *In-vivo* Imaging System. Scrb, scramble shRNA; sh#(1-2), shHMGA2#(1-2). Scale bar: 1mm.

The high expression of HMGA2 in glioma-initiating cells/glioma stem cells/tumor propagating cells (GICs/GSCs/TPCs) prompted us to examine whether HMGA2 is essential for GIC propagating (self-renewal) and tumorigenicity. To this end, we established a few GIC lines from surgical resected high-grade glioma samples using free-floating neurosphere or adherent cultures. These GICs ubiquitously express stem-like biomarkers (SOX2 and NESTIN), can self-renew in the presence of mitogens (fibroblast growth factor and epidermal growth factors), and give rise to a variety of differentiated progenies such as neurons, astrocytes and pericytes (Figure 2C and Supplementary Figure S2E-S2G). Moreover, they are capable of generating intracranial tumors when xenografted into immune-compromised nude mice (Figure 3E and Figure 7G).

Having the tools ready, we first asked whether HMGA2 is essential for GIC self-renewal. We prepared shRNA-expressing lentiviruses to target the expression of *HMGA2*. Among them, shHMGA2#1 and shHMGA2#2 can efficiently down-regulate HMGA2 mRNA and protein levels in U251 cells (Supplementary Figure S3A-S3C). We therefore transduced them into three GIC lines, TPC1115, TPC0411 and TPC0209. In both adherent (Figure 3A) and free-floating neurosphere (Figure 3B-3C) culture conditions, *HMGA2* knockdown leads to compromised propagating capabilities in all tested GICs. Furthermore, when GICs transduced with shHMGA2 lentiviruses were xenografted intracranially into striata of athymic nude mice, they produced much smaller tumors compared to GICs expressing scramble control shRNAs (Figure 3E and Figure 7G). In contrast, *HMGA2* knockdown has minimal effects on proliferation of U251 and U87MG glioma cells, which is exemplified by much milder changes in MTT assays, ratios of Ki67-positive cells and BrdU incorporation (Figure 3D, Supplementary Figure S3D-S3F and data not shown). This indicated that HMGA2 preferentially sustains GIC self-renewal. We'd like to point out that *HMGA2*-depleted U251 cells also displayed compromised subcutaneous tumorigenicity, suggesting HMGA2 roles in promoting tumorigenesis *in vivo* (Supplementary Figure S3G-S3H).

### HMGA2 potentiates pericyte differentiation and invasive properties of glioma-initiating cells

**Figure 4:**
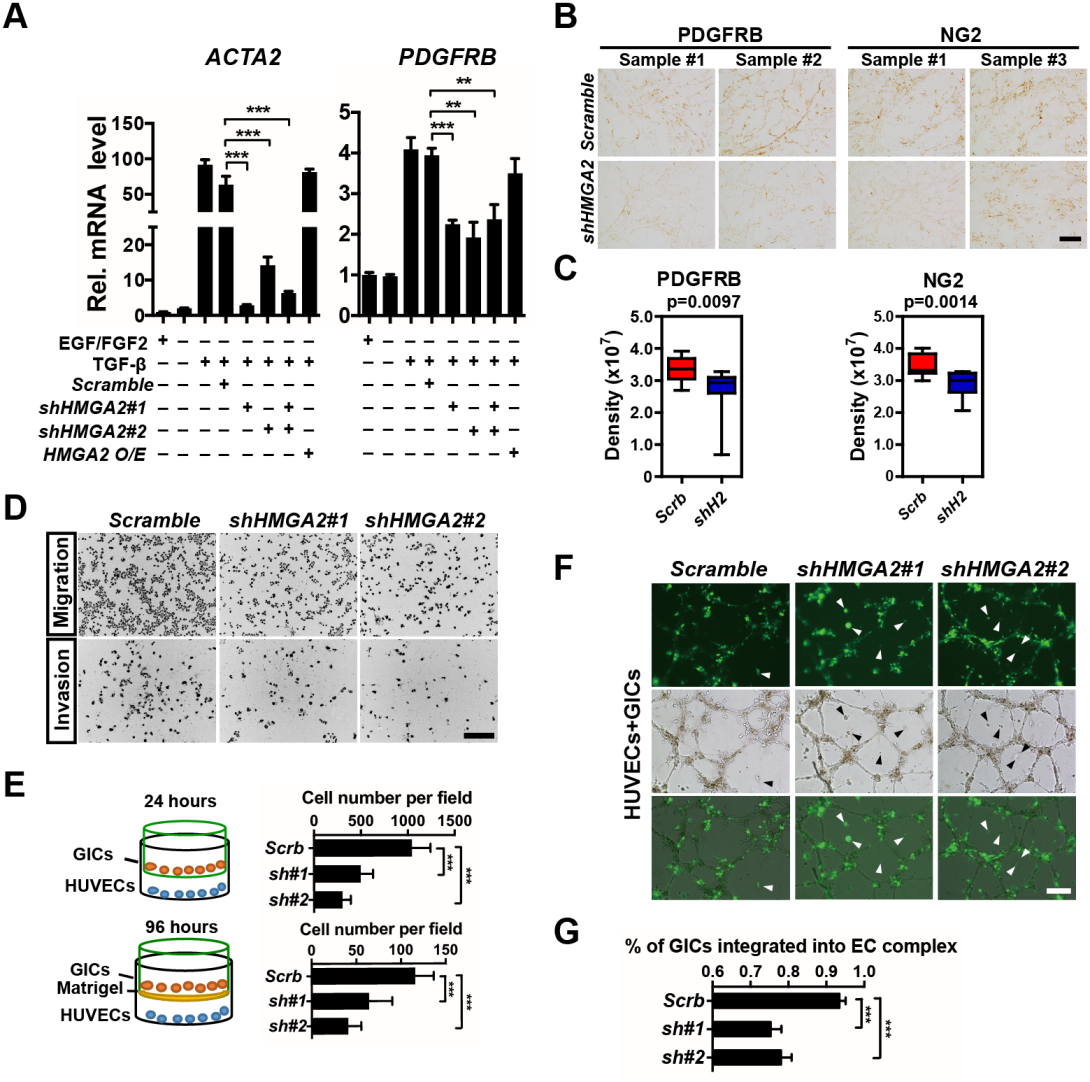
HMGA2 is required for invasive properties, pericyte differentiation, and EC integration of glioma-initiating cells **A.** Quantification and statistical analyses of *ACTA2* (α-SMA) and *PDGFRB* expression levels in TPC1115 GICs upon indicated treatments. **B.** Representative images showing immunohistochemistry of PDGFRB and NG2 in GICs/matrigel transplants transduced with scramble- and shHMGA2-lentiruses. **C.** Quantification and statistical analyses of PDGFRB and NG2 expression in B (n=4 samples). Measuring and quantifications of IHC images were performed using the Image-pro Plus 6.0 software (Media Cybernetics). **D.** Representative images showing migratory (top) and invasive (bottom) TPC1115-derived cells transduced with indicated lentiviruses in transwell assays. Trans-welled Cells were stained with DAPI for counting. **E.** Quantification of migratory (top) and invasive (bottom) GIC-derived cells in three independent experiments. **F.** HUVECs were plated onto matrigel to form endothelial cell (EC) complexes for 16-18 hours. Next, transduced TPC1115 GICs (ZsGreen+) were plated onto EC complexes and co-cultured for 24 hours. Top: cell derived from lentiviral transduced GICs; middle: phase-contrast images; bottom: merged images. Arrowheads point to unincorporated GICs. **G.** Quantification and statistical analyses of GIC integration into EC complexes. Scrb, scramble shRNA; sh#(1-2), shHMGA2#(1-2). Scale bars: (B) 100 μm, (D) 200 μm, (F) 300 μm.

We next studied HMGA2′s function in promoting mesenchymal phenotypes and invasiveness in gliomas. We first explored whether HMGA2 is crucial for pericyte differentiation of GICs induced by cytokines. TGF-β treatment greatly enhanced expression levels of *ACTA2* (α-SMA coding gene) and *PDGFRB* (platelet-derived growth factor receptor, beta polypeptide), two pericyte biomarkers in TPC1115 GICs (Figure 4A and Supplementary Figure S2F). Consistent with previous studies, the expressions of *HMGA2* [36], and *NG2* (another pericyte marker), were also moderately elevated upon TGF-β treatment (Supplementary Figure S4A). TNF-α treatment has similar effects on expressions of pericyte markers (Supplementary Figure S4A). However, treating GICs with PDGFBB, one of the ligands for PDGFRB, has minimal effects (Supplementary Figure S4A). Next, we knocked down expression of *HMGA2* during GIC differentiation elicited by TGF-β, and found the elevated expressions of *ACTA2* and *PDGFRB* were largely abrogated (Figure 4A). Furthermore, the expression densities of PDGFRB and NG2 were much lower in cells derived from HMGA2-depleted GICs that were inoculated subcutaneously along with the matrigel matrix (Figure 4B-4C). Similarly, the expression levels of key mesenchymal molecules including *YKL40*/*CHI3L1* and *FN1* (*FIBRONECTIN 1*) in GICs can be greatly induced by TNF-α treatment or overexpressing C/EBPβ, a transcription factor essential in mesenchymal transformation of gliomas (Supplementary Figure S4A-S4B). TGF-β treatment also enhanced the expression of *FN1* (Supplementary Figure S4A). We next explored whether HMGA2 is crucial in maintaining expression of these molecules during mesenchymal differentiation. As expected, depleting of *HMGA2* leads to significant downregulation of *YKL40*, *FN1* and *SNAIL2*/*SLUG* in TNF-α, TGF-β and C/EBPβ induced differentiation of TPC1115 GICs (Supplementary Figure S4C-S4E).

We next studied whether HMGA2 is required for migration and invasion of GIC-derived cells. In transwell migration assays, human umbilical vein endothelial cells (HUVECs) were plated on the bottom surface of Boyden chambers, and GICs onto the insert membranes with the presence of TGF-β. In invasion assays, matrigel was coated on the top of the insert membrane prior to GIC plating. *HMGA2*-depleted GICs were much less potent in both migration and invasion (Figure 4D-4E). HUVECs can form vessel-like tubular structures (EC complex) *in vitro* (Figure 4F, Supplementary Figure S4F). Co-cultured GICs were mostly integrated into the complex in 24 hours post-plating (Supplementary Figure S4F). However, in accordance with transwell assays, *HMGA2*-depleted GICs were less capable of integrating into the EC complex than control GICs (Figure 4F-4G). Furthermore, xenografted brain tumors derived from *HMGA2*-depleted GICs contained much fewer vascular structures (Figure 7G, Supplementary Figure S6H). Collectively, HMGA2 is essential for chemotactic, invasive and angiogenic potentials of GIC-derived cells.

### HMGA2 is required for expression of genes essential for GIC cell-cycle progression and invasion

**Figure 5:**
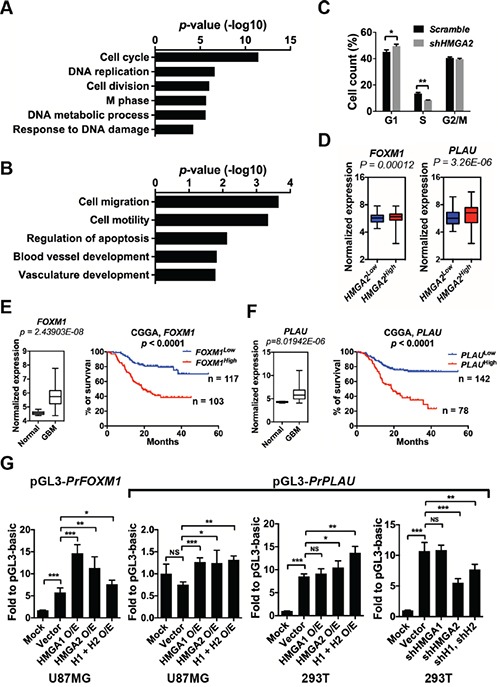
HMGA2 facilitates the expressions of *FOXM1* and *PLAU* **A.** Gene Ontology (GO) analyses of commonly down-regulated genes in TPC1115 GICs depleted with *HMGA2*, *HMGA1* and *HMGA2/HMGA1*. **B.** List of GO terms summarizing down-regulated genes in TPC1115 GICs depleted with *HMGA2*. **C.** Cell cycle changes of TPC1115 GICs upon knockdown of *HMGA2*. **D.** Box plots showing normalized expressions of *FOXM1* and *PLAU* metagene in *HMGA2*^Low^ and *HMGA2*^High^ GBMs using data retrieved from TCGA. P values were determined using a nonparametric Wilcoxon test. *HMGA2*^low^ (n = 471) and *HMGA2*^high^ (n = 87) expression groups were defined using the minimum P-value approach (maximal chi square statistics approach). **E-F.** Left: expression levels of *FOXM1* (E) and *PLAU* (F) in 10 normal brains and 557 GBM samples using data retrieved from TCGA; right: Kaplan-Meier survival plots showing correlations of glioma patients' survival with expression levels of *FOXM1* (E) and *PLAU* (F) using the CGGA (Chinese Glioma Genome Atlas) dataset. **G.** Dual-luciferase assays showing relative luciferase activities of *FOXM1* and *PLAU* promoter fragments (*PrFOXM1* and *PrPLAU*) in U87MG and 293T cells transfected with indicated vectors. O/E, overexpression; H1, HMGA1; H2, HMGA2.

To understand molecular signaling regulated by HMGA2, we performed RNA-seq transcriptome analysis of TPC1115 GICs tranduced with shRNAs against *HMGA1* or *HMGA2* or both for 72 hours in the absence of EGF/FGF2 mitogens. Since HMGA1/2 promotes loosening of chromatin, thus facilitates gene expression, we focused on genes down-regulated upon *HMGA2* knockdown (KD). Consistent with reduced proliferation of GICs upon *HMGA2* (Figure 3A-3C) or *HMGA1* knockdown (data not shown), down-regulated transcripts shared in *HMGA1*-KD, *HMGA2*-KD and *HMGA1*/*HMGA2*-KD GICs are enriched with those encoding key regulators for cell-cycle progression (Figure 5A, Supplementary Table S1A). Twenty-four out of fifty-six transcripts in this group were categorized into genes involved in cell cycle regulation, including *CCND1*, *CCNE2*, *MCM2*, *MCM6*, *E2F1*, *DSN1*, *ASPM*, *BIRC5*, *SKA3* and *THBS1* (Supplementary Table S1). Consistently, cell-cycle analysis of *HMGA2*-depleted GICs showed more cells were at G1 phase and fewer cells at S phase relative to scramble-transduced GICs (Figure 5C). However, we reasoned that these cell-cycle regulators are unlikely to be HMGA2/HMGA1 direct targets, because *HMGA2* depletion in immortal cells (e.g. U251 and U87MG) has neglected effects on cell proliferation (Figure 3D, Supplementary Figure S3D-S3F). Surprisingly, the *SOX2* expressions were not altered after *HMGA2* knockdown or overexpression. HMGA2 has also been reported to promote *SOX2* expression by directly binding to the *SOX2* promoter in GBM cells [35]. This discrepancy might be due to heterogeneity between tumors.

One of the molecules, *FOXM1* (Foxhead Box M1), is an attractive candidate that may be a downstream target of HMGA2. First, *FOXM1* expression levels are positively correlated with *HMGA2* expression in GBM specimens from the TCGA dataset and glioma samples from the CGGA dataset (Figure 5D; Supplementary Figure S5A, the left panels). Second, *FOXM1* is overrepresented in GBM patients and its expression levels are inversely correlated with glioma patients' survival period using the CGGA dataset [34] (Figure 5E). Last, FOXM1 stimulates self-renewal, tumorigenicity and invasiveness of glioblastoma stem-like cells by transactivating a number of molecules, including *IPO7* (importing-7), *CDC20*, *PDGF-A*, *ANXA1*, *MMP-2* and *VEGF* [37–41]. FOXM1 also promotes β-catenin nuclear localization and controls Wnt target-gene expression during glioma tumorigenesis [42].

Moreover, the transcripts specifically down-regulated in HMGA2-depleted GICs are enriched with molecules implicated in cytoskeleton, cell migration, cell motility, and blood vessel development, including genes encoding ADAM9 (ADAM metallopeptidase domain 9), ROCK1 (Rho-associated, coiled-coil containing protein kinase 1), KIF4 (kinesin family member 4), PLAU/u-PA (plasminogen activator, urokinase), ITGA4 (integrin, alpha 4), CYR61 (cysteine-rich, angiogenic inducer, 61) (Figure 5B, Supplementary Table S1B). Among them, PLAU, a serine protease involved in degradation of the extracellular matrix and tumor cell migration and proliferation, might mediate migration and invasion of U87MG glioma cells [43]. Similarly, *PLAU* is highly expressed in GBM specimens (Figure 5F), and its expression level is positively correlated with *HMGA2* expression in GBM specimens and inversely correlated with glioma patients' survival period (Figure 5D, 5F; Supplementary Figure S5A, the right panels).

Using qRT-PCR, we confirmed that the expression levels of *FOXM1*, *PLAU*, *CYR61*, *THBS1* and *ADAM9* are indeed significantly down-regulated upon *HMGA2* depletion in TPC1115 GICs, U87MG glioma cells and SK-N-SH neuroblastoma cells (Supplementary Figure S5B-S5D). Consistent with the transcriptome analysis, depletion of *HMGA1* in these cells has minimum or opposite effects on the expressions of *PLAU*, *CYR61* and *ADAM9* (Supplementary Figure S5B-S5D).

Next, we carried out luciferase reporter assays to explore whether HMGA2 can facilitate the expression of *FOXM1* and *PLAU*. The promoter region of *FOXM1* (−2019 to +57 relative to the transcription starting site/TSS), and *PLAU* (−1886 to +26 relative to the TSS) were cloned into pGL3-Basic luciferase reporter vector. In U87MG glioma cells, overexpression of either HMGA1 or HMGA2 or both significantly enhances luciferase activities driven by the two promoters. In 293T cells, there was robust basic luciferase activity driven by the *PLAU* promoter. Knocking down or overexpressing HMGA2 significantly reduces or enhances the luciferase activities driven by the *PLAU* promoter respectively, whereas manipulating the expression of HMGA1 has no effect (Figure 5G).

### Expression of FOXM1 or PLAU in HMGA2-depleted GICs rescued their defects in self-renewal, invasion and tumorigenicity

**Figure 6:**
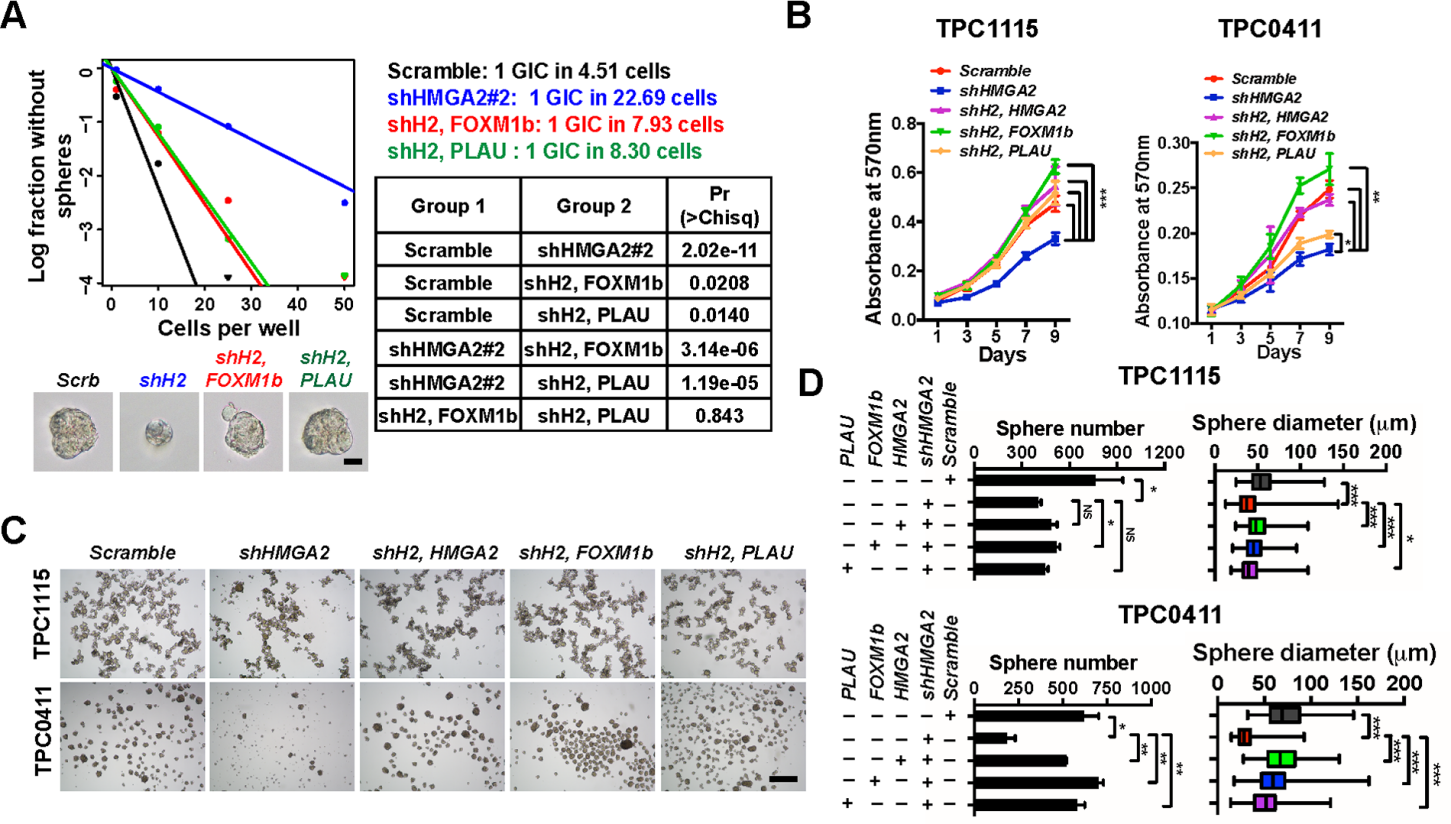
HMGA2 relies on FOXM1 and PLAU to maintain GIC self-renewal **A.** TPC1115 GICs were transduced with indicated lentiviruses before plating into 96-well plates for limiting dilution assays. Cultures were maintained until day 10, when the number of wells containing spheres for each cell plating density (number of positive cultures) was recorded, calculated and plotted using online ELDA analysis program. Bottom, representative sphere images in each group. Right, incidence of sphere-forming glioma initiating cells (GICs), and P values [Pr(>Chisq)] between groups. **B.** MTT assay showing effects of *HMGA2* knockdown on GIC cell propagation in adherent culture conditions. **C.** Representative images showing TPC1115 and TPC0411 GICs cultured in neurosphere conditions for 7 days after transducing with indicated lentiviruses. **D.** Quantification of sphere numbers and diameters of three independent experiments in (C). shH2, shHMGA2#2. Scale bars: (A) 50 μm; (C) 500 μm.

**Figure 7:**
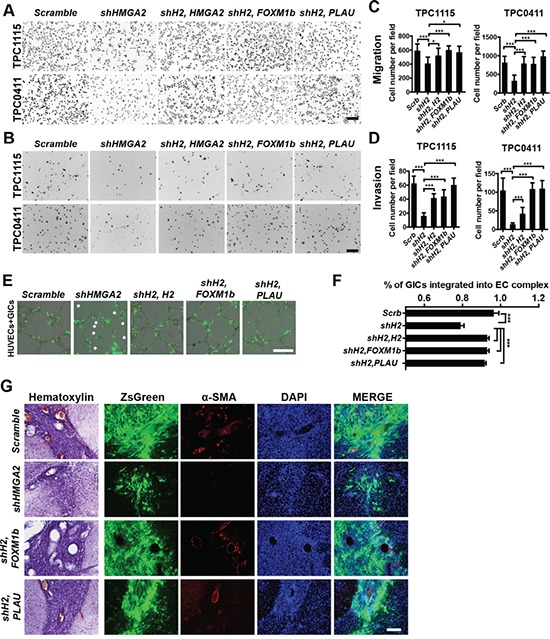
Overexpression of FOXM1 or PLAU restores invasive, tumorigenic and angiogenic potentials in HMGA2-depleted GICs **A-B.** Representative images showing migratory (A) and invasive (B) TPC1115- and TPC0411-derived cells transduced with indicated lentiviruses in transwell assays. Trans-welled Cells were stained with DAPI for counting. **C-D.** Quantification of migratory (C) and invasive (D) GIC-derived cells in three independent experiments. **E.** Representative images showing integration of transduced GICs (ZsGreen+) with EC complexes. Arrowheads point to unincorporated GICs. **F.** Quantification and statistical analyses of GIC integration into EC complexes. **G.** Representative immuohistochemical and immunofluorescent images showing sections from brains implanted with TPC1115 GICs transduced with indicated lentiviruses (ZsGreen expression) and stained with α-SMA and DAPI. shH2, shHMGA2#2; H2, HMGA2. Scale bars: (A-B) 200 μm; (E) 300 μm; (G) 100 μm.

To elucidate whether these candidates are responsible for HMGA2′s roles in GIC self-renewal and invasive properties, we carried out functional rescue experiments. In limiting dilution assays (Figure 6A), adherent (Figure 6B) and neurosphere cultures (Figure 6C-6D, Supplementary Figure S6E-S6G), overexpression of FOXM1 or PLAU mostly reversed the proliferative defects of TPC0411 GICs caused by HMGA2 knockdown in both the first and second passages (Supplementary Figure S6F). Moreover, FOXM1 or PLAU overexpression can partially rescue migratory and invasive potentials in *HMGA2*-depleted GICs, which is similar to putting back shRNA-resistant *HMGA2* (Figure 7A-7D, Supplementary Figure S6A). Similarly, re-expression of HMGA2, FOXM1 or PLAU largely reversed defects in EC complex integration of HMGA2-depleted GICs (Figure 7E-7F). The *FOXM1* gene transcribes several transcript variants encoding different isoforms. Intriguingly, it's the expression of the transcript variant 3 of *FOXM1* (*FOXM1v3*/*FOXM1b*, NM_202003.2), but not *FOXM1v2*/*FOXM1c* (NM_021953.3) that is more prominently down-regulated upon HMGA2 knockdown (Supplementary Figure S6B). Accordingly, it was the overexpression of *FOXM1b* but not *FOXM1c* that restored the self-renewal and invasiveness potentials in HMGA2 depleted GICs (Supplementary Figure S6C-S6D), which coincides with previous studies [44]. Finally, expression of FOXM1b or PLAU in *HMGA2*-depleted GICs largely reverted their tumorigenic potentials when transplanted intracranially. Histological and immunofluorescent analyses showed restored cellularity and tumor neo-angiogenesis in FOXM1b- or PLAU-expressing GICs that were devoid of *HMGA2* (Figure 7G and Supplementary Figure S6H).

To summarize, we showed FOXM1 and PLAU are two key downstream targets of HMGA2 to maintain GIC propagation, gliomagenesis and aggressiveness both *in vitro* and *in vivo*.

## DISCUSSION

In this study, we unveiled that HMGA2 is highly expressed in mesenchymal GBMs, labels glioma-initiating cells (GICs) and glioma pericytes. Furthermore, HMGA2 is essential for GICs' potentials of self-renewal, tumorigenecity, mesenchymal and pericyte differentiation. Using transcriptome analyses and rescuing experiments, we also characterized *FOXM1* and *PLAU* as two direct target genes of HMGA2. Future studies will elucidate whether targeting HMGA2-mediated signaling would improve survivals in GBM genetic mouse models.

Although HMGA2 depletion has little effects on proliferation of regular glioma cells, it's essential for self-renewal of GICs. Previous studies have proposed several mechanisms that may account for HMGA2 abilities to promote stem/progenitor cell self-renewal and tumor cell transformation. For example, HMGA2 promotes NSC self-renewal by negatively regulating *Ink4a*/*Arf* expression from the *CDKN2A* locus [45]. Moreover, HMGA2 may control expression from this locus indirectly by repressing the expression of *JunB*, whose gene product is a component of AP1 complex and an activator of *Ink4a*/*Arf* expression, in neoplastic transformation of thyroid cells [46]. However, the expressions of *JUNB*, *INK4A* and *ARF* are unaltered in HMGA1 and/or HMGA2 depleted GICs, suggesting GICs utilize distinct mechanisms directed by HMGA2.

Utilizing transcriptome profiling and functional rescuing assays, we demonstrated that HMGA2 sustains self-renewal and invasiveness of GICs by allowing expression of *FOXM1* and *PLAU*, two core players that have been implicated in gliomagenesis and aggressiveness. FOXM1 is a transcriptional activator involved in cell proliferation. It is phosphorylated in M phase and induces the expression of several cell cycle genes, such as cyclin B1 and cyclin D1 [47–49]. PLAU (uPA), via signals through its receptor PLAUR (uPAR), exerts multiple functions not only in invasion of migrating cells by digesting extracellular molecules, but also in cellular adhesion, differentiation, proliferation and migration in a non-proteolytic fashion [50]. The expression of *PLAU* is drastically reduced in *HMGA2*- but not *HMGA1*-depleted GICs. Further, overexpression of PLAU largely rescued self-renewal, migration, invasion and vascular formation defects induced by *HMGA2* knockdown. Interestingly, FOXM1 transactives *PLAUR* expression to promote colon cancer progression and metastasis [51]. These suggest the presence of the HMGA2-FOXM1/PLAU-PLAUR axis in maintaining GIC self-renewal and aggressiveness.

HMGA2 is expressed in both GICs and pericytes (perivascular mural cells). We and other groups found GICs are able to generating pericytes in a xenografted GBM model or in culture by applying TGF-β [21]. We also revealed HMGA2′s essential roles in this process, as depletion of HMGA2 mostly abolishes GICs' potential of pericyte differentiation. On the other hand, a subgroup of pericytes are mesenchymal stem cells (MSCs) capable of differentiating into multiple cell types, including osteoblasts, fibroblasts, adipocytes, myogenic cells and odontoblasts [52, 53]. Therefore, pericytes in gliomas could be stem-like cell pools and it would be tempting to explore if HMGA2 is key to regulating the fate switch between the two cell populations.

## MATERIALS AND METHODS

### Glioma specimen and brain tissue collection

Glioma surgical specimens were collected in Wuhan Union Hospital and The Tenth Affiliated Hospital, Tongji University in accordance with institution-approved protocols. All patients signed and approved consent forms prior to surgery. Collected specimens were split into three parts for protein/RNA isolation, 4% paraformaldehyde (PFA) fixation/cryo-sectioning and GIC establishment (see below sections) respectively. Specimens were examined by neuropathologists to verify tumor types and grades.

### Cell culture

U251 and U87MG glioma cells were gifts from Dr. Xiaozhong Peng at Peking Union Medical College. SK-N-SH neuroblastoma cells were purchased from China Center for Type Culture Collection. Cells were maintained in according culture media (DMEM or MEM) containing 10% fetal bovine serum (Life Technologies, Grand Island, NY, USA).

### Analysis of glioma patients' survival and expression data

Tumor gene expression and survival data of glioma patients were retrieved from The Cancer Genome Atlas (TCGA) and The Chinese Glioma Genome Atlas (CGGA). Patients were divided into *HMGA2*^low^ and *HMGA2*^high^ expression groups using the minimum P-value approach (maximal chi square statistics approach) or by the median expression levels.

### Establishment, propagating and differentiation of glioma-initiating cells (GICs)

Surgically removed GBM specimens were washed with and minced in filter-sterilized hibernation buffer (30 mM KCl, 5 mM NaOH, 5 mM NaH_2_PO_4_, 5.5 mM glucose, 0.5 mM MgCl_2_, 20 mM Na-pyruvate, 200 mM Sorbitol, pH 7.4) followed by dissociating into single cells using pre-warmed Papain (Worthington Biochemical, Lakewood, NJ, USA) enzyme solution (1×DMEMD, 1 mM Na-pyruvate, 1 mM L-Glutamine, 1 mM N-Acetyl-L-Cysteine, 20 U/mL Papain, 12 μg/mL DNase I). Dissociated cells were cultured in low-adhesion plates (Corning, NY, USA) using serum-free media consisting of DMEM/F12 media (Life Technologies), N2 and B27 supplements (1×, Life Technologies), 1 mM Na-pyruvate, 1 mM L-Glutamine, 1 mM N-Acetyl-L-Cysteine (NAC), human recombinant FGF2 and EGF (20 ng/mL each; Life Technologies) to form neurospheres. Fresh N2, B27, NAC, FGF2 and EGF supplements (10×) were added every 3-4 days. Alternatively, dissociated GBM cells (1×10^6^ cells per 10cm plate) were plated onto laminin (L2020, Sigma-Aldrich, St. Louis, MO, USA) coated plates using aforementioned serum-free media. Medium was replaced every 3-4 days. Floating or adherent GIC cells were passed every 7 days. We tested the expression of stemness proteins such as SOX2 and NESTIN and the differentiation capability at the 3rd passage using immunofluorescent assays. Differentiation of GICs was achieved by removing FGF2 and EGF from the medium and adding 1-10% FBS, 5ng/mL TGF-β or 5ng/mL TNF-α for 6-7 days. For mesenchymal differentiation, GICs were transduced with lentiviruses expressing *C/EBP*β and further cultured for 6 days. GICs between passages 7 and 12 were used in all studies. For neurosphere formation assays, GICs were grown in 6-well low-adhesion plates (3×10^4^ cells per well) for 6-7 days followed by neurosphere measurement and cell number counting.

### Preparation of lentiviruses for overexpression and shRNA-mediated knockdown

Annealed DNA fragments containing scrambled sequences and short-hairpin RNA (shRNA) sequences against *HMGA1* and *HMGA2* were cloned into a modified pLKO.1 lentiviral vector in which the puromyin-resistant cassette is replace with an ZsGreen-expressing cassette. *HMGA1*, *HMGA2*, *FOXM1* and *PLAU* cDNAs were cloned into pCDH lentiviral vectors for overexpression. For production of lentiviruses, pLKO.1 or pCDH vectors were co-transfected into 293T cells with psPAX and pMD2.G packaging plasmids using calcium-phosphate methods. To prepare viruses used in GIC culture, regular 293T culture medium was replaced with serum-free GIC medium (see above section) 24 hours after transfection; and supernatants were collected 24 hours later. ShRNA targeting sequences: shHMGA1 (5′-CAA CTC CAG GAA GGA AAC CAA-3′); shHMGA2#1 (5′-AGT CCC TCT AAA GCA GCT CAA-3′); shHMGA2#2 (5′-GCC CAA GGC ACT TTC AAT CTC-3′).

### Cell proliferation assay (MTT assay) and cell cycle analysis

GICs were transduced with indicated lentiviruses. Forty-eight hours later, 1000 cells were plated into 96-well plates with the presence of 5 μL MTT solutions (5 mg/mL) in each well. Cell proliferation assays were performed on days 1, 3, 5, 7 and 9 post-plating using the classical MTT reduction assay. Briefly, cells were added with 100 μL dissolving solutions (50% dimethylformamide, 30% SDS, pH4.7), and were incubated for 4 hours at 37°C. The absorbance was detected at 570 nm with a Versamax Microplate Reader (Molecular Devices, Sunnyvale, CA, USA).

For cell cycle analysis, ethanol-fixed cells were treated with RNase A and stained with propidium iodide (100 μg/mL in PBS) at 4°C for 15 minutes. The stained cells were detected using CyAn-ADP flow cytometer (Beckman Coulter, Brea, CA, USA). Data were analyzed using a cell-cycle analysis program (Flowjo 7.6) to calculate percentiles of cells at G1, G2/M and S phases.

### *In vitro* limiting dilution assay

GICs were transduced with lentiviruses. Forty-eight hours later, viable cells were counted and decreasing numbers of cells per well (50, 20, 10 and 1) were plated into 96-well plates. Cultures were fed 25μl of medium every 3 days until day 10, when the number of positive cultures was recorded. Extreme limiting dilution analyses were carried out using a web-based software available at http://bioinf.wehi.edu.au/software/elda/ [54]. Regression lines were plotted and x-intercept values calculated, which represent the number of cells required to form at least one tumor sphere in every well.

### *In vitro* endothelial cell (EC) complex formation

Human umbilical vein endothelial cells (HUVECs) were gifts from Dr. Luyang Yu at Zhejiang University and were maintained in EBM-2 medium (LONZA, Basel, Switzerland) at 37°C and 5% CO_2_. HUVECs between passages 7 and 10 were used for EC formation. HUVECs (2.0×10^4^cells per well) were plated onto 48-well plates coated with Matrigel (125 μL per well, BD Biosciences, Franklin Lakes, New Jersey, USA) in the presence of 2-5 ng/mL TGF-β. 16-18 hours later, tubule or mesh-like structures were formed. Then lentivirus-transduced GICs (2.0×10^4^) were seeded onto the preformed EC complex. After 24 hours incubation, EC-GIC cultures were fixed, imaged, and quantified.

### Transwell migration and invasion assays

HUVECs (4.0-5.0×10^4^ cells per well for migration assays; 1.0-2.0 × 10^4^ cells per well for invasion assays) were pre-cultured in 24-well plates for 16-18 hours. Dissociated GICs were seeded into Boyden chambers (8 μm pore size with polycarbonate membrane) supplemented with 2 ng/ml TGF-β. The chambers were coated with Matrigel Matrix (354234, BD Biosciences) for invasion assays. After 24 hours (migration) or 96 hours (invasion), cells on the top surface of the insert were removed by wiping with cotton swabs. Cells that migrated to the bottom surface of the insert were stained in DAPI and subjected to microscopic inspection. Images of five random fields were captured from each membrane, and then numbers of migratory or invasive cells was counted.

### Immunohistochemistry (IHC), immunofluorescence (IF), and immunoblotting

Paraffin-embedded glioma tissue arrays were purchased from Alenabio (Xi'an, China). Sections were baked in oven at 60°C for 30 minutes on a vertical rack to melt the extra layer of coated paraffin. Then slides were immersed in xylene twice, rehydrated in graded concentrations of ethyl alcohol, and then washed in dH_2_O. Then slides were incubated in 10 mM sodium citrate (pH 6.4) retrieval solution and heated at 95°C for 15 minutes. Slides were then sequentially cooled, washed in phosphate-buffered saline for 10 minutes, and incubated in 0.3% H_2_O_2_ for 15 minutes to deplete endogenous peroxidase activity. The tissue arrays were blocked with blocking buffer (3% heat-inactivated normal goat serum, 0.1% bovine serum albumin and 0.1% Triton-X 100 in 10 mM Tris, 100 mM NaCl, pH 7.4) for at least 1 hour. Sections were then incubated overnight with the anti-HMGA2 antibody diluted in blocking buffer. The next day, the staining was revealed using 3,3′-diaminobenzidine (DAB) as the substrate after peroxidase-conjugated avidin/biotinylated enzyme complex incubation (VECTASTAIN Elite ABC system, Vector Labs, Burlingame, CA, USA). Measuring and quantifications of IHC images were performed using the Image-pro Plus 6.0 software (Media Cybernetics). For immunofluorescent stainings, 4% paraformaldehyde (PFA) fixed 14 μm cryo-sections or cells were permeabilized and blocked with blocking buffer for one hour at R/T. Samples were then incubated with primary antibodies diluted in blocking buffer overnight at 4°C or at R/T. The next day, slides were washed 3 times for 10 minutes with 1 × PBS and incubated with second antibodies in blocking buffer at R/T for an hour. Slides were mounted with anti-fade solution with DAPI after PBS-wash for three times. For BrdU (5-bromo-2′-deoxyuridine) IF stainings, BrdU was applied to culture media to a final concentration of 3 μg/mL three hours before fixation. Post-fixed cells were treated with 2 mol/L HCl for 15 minutes before blocking. All immunofluorescent images comparing expression levels were acquired at equal exposure times. Immunoblotting assays were carried out according to standard procedures. The following primary antibodies were used: human SOX2 (IF, 1:200, AM2048A, Abgent, Suzhou, China), HMGA1 (IF/IHC, 1:800; WB, 1:10000, ab129153, Abcam, Cambridge, MA, USA), HMGA2 (IF/IHC, 1:400; WB, 1:3000, ab97276, Abcam), NESTIN (IHC, 1:200, SC-23927, Santa Cruz, CA, USA), CD133 (IHC, 1:200, 293C3, Miltenyi Biotec, Bergisch Gladbach, Germany), α-SMA/ACTA2 (IF, 1:200, a gift from Dr. Hongliang Li), Ki67 (IF/IHC, 1:400, #9449, Cell Signaling, Danvers, MA, USA), IBA-1 (IHC, 1:400, ab5076, Abcam), β-catenin (IHC, 1:200, 610153, BD Biosciences), CD44 (IHC, 1:200, 550392, BD Biosciences). CD146 (IHC, 1:100, 550315, BD Biosciences), CD248 (IHC, 1:100, 564994, BD Biosciences), PDGFRB (WB, 1:2000, sc-432, Santa cruz), PDGFRB/CD140b (IHC 1:100, 564994, BD Biosciences), CD34 (IHC, 1:100, 555821, BD Biosciences), FLAG (WB, 1:10000, F1804, Sigma-Aldrich). The following secondary antibodies were used: anti-rabbit, anti-rat, anti-goat and anti-mouse conjugated to Alexa Fluor 488, Alexa Fluor 555 and Alexa Fluor 647 (IF, 1:1000, Life Technologies), or to biotin (IHC, 1:200, Jackson ImmunoResearch, West Grove, PA, USA), or to HRP (WB, 1:5000, Jackson ImmunoResearch).

### Matrigel angiogenesis assay

Suspended 1×10^5^ lentivirus-transduced GICs were mixed with 200 μL Matrigel Matrix at a volume ratio of 1:1 on ice. The mix was injected subcutaneously to the back of 6-week-old male Balb/c athymic nude mice to form plugs (n = 4 samples for each group). Fourteen days after injection, the Matrigel plugs were dissected out and freshly cryosectioned at the thickness of 5 μm. Sections were immunohistochemically stained with anti-PDGFRB and anti-NG2 antibodies.

### Subcutaneous xenografting of U251 glioma cells and intracranial xenografting of GICs

All animal procedures were approved by the Animal Care and Ethical Committee of College of Life Sciences at Wuhan University. Xenografting experiments were carried out using 6-week-old male Balb/c athymic nude mice (HNSJA, Changsha, China). Mice were housed in a certified specific-pathogen-free (SPF) facility. U251 glioma cells were transfected with lentivirus (moi≈10) expressing scramble control or HMGA2 shRNAs 48 hours before inoculation. A total of 1×10^6^ suspended cells (in 1×PBS) were inoculated subcutaneously in each side of the anterior lateral thoracic wall. Tumor dimensions were measured every week and animals were sacrificed 4 weeks after inoculation. A total of 1×10^5^ lentivirus-transduced GICs (in culture medium) were injected intracranially using a stereotactic device (RWD) and a Hamilton syringe at a depth of 2.5 mm into the right striata of cerebral hemisphere [55]. Animals were 8 weeks post-surgery or when they showed significant signs of tumor formation (tremor, hunching or seizure). Xenografted nude mice were transcardiac perfused with 4% PFA and brains were dissected out. Fluorescent images of brains were captured using the Maestro *In-vivo* Imaging System (PerkinElmer, Waltham, MA, USA).

### RNA-seq transcriptome analysis

TPC1115 glioma-initiating cells were transduced with shRNAs against HMGA1 or HMGA2 or both (moi≈10). Cells were collected 72 hours post-transfection and subjected to RNA extraction using Trizol solution (Life Technologies) and the integrity of RNAs was analyzed using Agilent Bioanalyzer 2100. Library construction and paired-end sequencing were performed in Berry Genomics using Hiseq 2500. Quality control, reads alignment and gene-expression analysis were also carried out in Berry Genomics. Clean reads were mapped to the human genome using the TopHat software. An R package, edgeR was applied for transcription quantification and differential expression analysis using a cutoff of P<0.05. Gene-ontology analysis was conducted using the Database for Annotation, Visualization and Integrated Discovery, a web-accessible program [56].

### Luciferase reporter assay

Luciferase reporter assays were conducted in U87MG and 293T cells using the Dual-Luciferase Reporter Assay System (Promega, Madison, WI, USA) according to manufacturer's manual with slight modifications. The promoter region of *FOXM1* (−2019 to +57 relative to TSS), and *PLAU* (−1886 to +26 relative the TSS) were amplified from genomic DNA of human fetal brain using KOD polymerase (Toyobo, Osaka, Japan) and cloned into pGL3-Basic luciferase reporter vector and confirmed by sequencing. Cells were transfected using the calcium phosphate transfection protocol or Lipofectamine 3000 (Life Technologies). Luciferase activity was determined 36 hours post transfection, cells were lysed with the passive lysis buffer and read the GloMax multidectection system (Promega) according to the manufacturer's instructions. Relative luciferase activity was determined by a ratio of firefly luciferase activity to control Renilla luciferase activity.

### Real-time quantitative reverse transcription PCR (qRT-PCR)

Total RNAs (1-2 μg) were reverse-transcribed at 42°C using PrimerScript™ Reverse Transcriptase (Takara Bio, Shiga, Japan). Then iTaq™ Universal SYBR^®^ Green Supermix (Bio-rad, Hercules, CA, USA) was employed to perform quantitative PCR on a CFX Connect™ Real-Time PCR Detection System (Bio-rad). Gene expressions were determined using the 2^−ΔΔCt^ method, normalizing to housekeeping genes *GAPDH* or *ACTB*. Oligo sequences for qRT-PCR: *GAPDH* (forward: 5′-AAT CAA GTG GGG CGA TGC TG-3′, reverse: 5′-TGG TTC ACA CCC ATG ACG AA-3′); *ACTIN* (forward: 5′-CTC TTC CAG CCT TCC TTC CT-3′, reverse: 5′-AGC ACT GTG TTG GCG TAC AG-3′); *HMGA1* (forward: 5′-ACT GGA GTC TCC TGT GGT GTG T-3′, reverse: 5′-AGT GCT ATT TCC CCT CCC TTC-3′); *HMGA2* (forward: 5′-CAC TTC AGC CCA GGG ACA AC-3′, reverse: 5′-GCC TCT TGG CCG TTT TTC TC-3′); *ACTA2* (forward: 5′-CAA TGA GCT TCG TGT TGC CC-3′, reverse: 5′-GCA AGG CAT AGC CCT CAT AGA-3′); *SOX2* (forward: 5′-CAC AAC TCG GAG ATC AGC AA-3′, reverse: 5′-CGG GGC CGG TAT TTA TAA TC-3′); *FOXM1* (forward: 5′-AGT AGT GGG CCC AAC AAA TTC AT-3′, reverse: 5′-CTT TTG GCA TCA TAG CTG GTT TG-3′); *CYR61* (forward: 5′-GGT CAA AGT TAC CGG GCA GT-3′, reverse: 5′-GGA GGC ATC GAA TCC CAG C-3′); *PLAU* (forward: 5′-TGT GAA GCT GAT TTC CCA CCG-3′, reverse: 5′-GCC TTG GAG GGA ACA GAC GAG-3′).

### Statistical analyses

Data were presented as the mean ± SD (standard deviation) unless otherwise indicated. Statistical significance was determined by unpaired Student's t test, and a P value of less than 0.05 was considered statistically significant and marked as ‘ * ’; a P values less than 0.01 or 0.001 was marked as ‘ ** ’ and ‘ *** ’ respectively.

## SUPPLEMENTARY FIGURES AND TABLE


