# Supplementary material for: HMGA2 sustains self-renewal and invasiveness of glioma-initiating cells
Source: Oncotarget. 2016 May 31;7(28):44365–80. doi: 10.18632/oncotarget.9744 (PMC5190103; doi:10.18632/oncotarget.9744)
Supplement: Supplementary file 1 [file oncotarget-07-44365-s001.pdf]

HMGA2 sustains self-renewal and invasiveness of glioma-initiating cells

SUPPLEMENTARY FIGURES AND TABLE

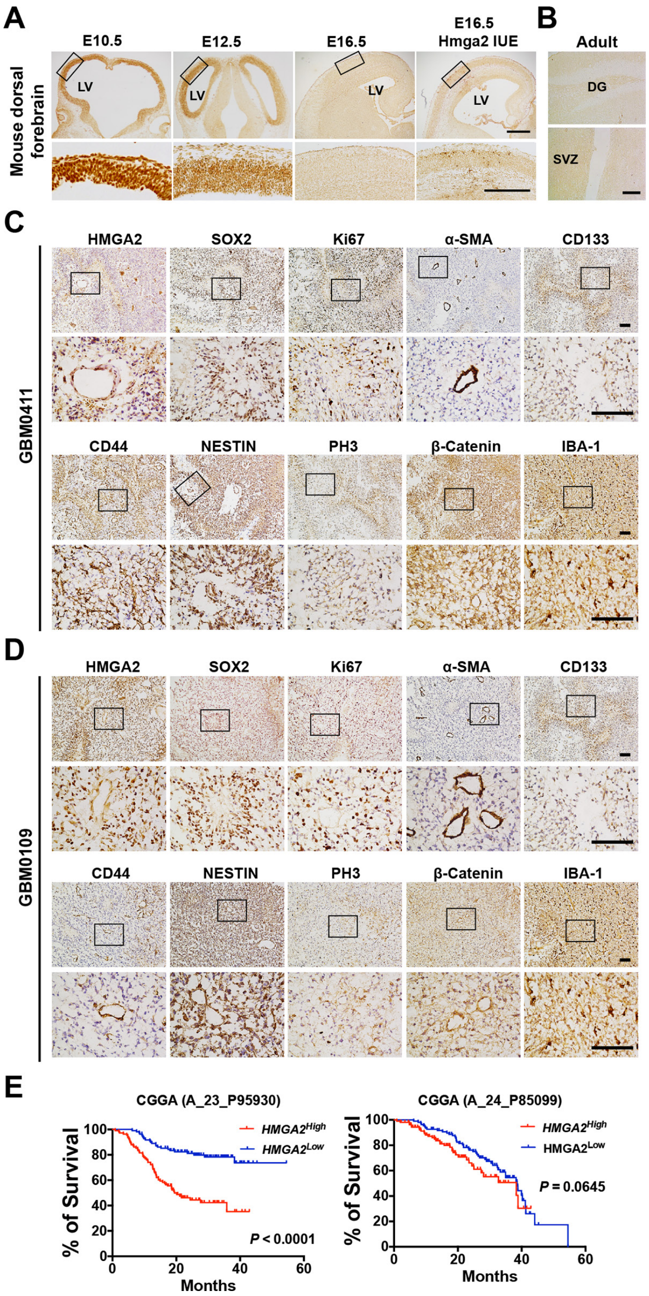

Supplementary Figure S1: HMGA2 is highly expressed in developing dorsal forebrain and glioblastoma (GBM) tissues. A-B. Immunohistochemistry staining of HMGA2 in developing mouse dorsal forebrains (A), and the subventricular zone (SVZ) and the dentate gyrus (DG) of adult mouse brain (B). HMGA2 expression is minimal in the E16.5 cortex (dorsal forebrain), adult SVZ and DG, whereas the anti-HMGA2 antibody specifically recognizes exogenously electroporated HMGA2. **C-D.** Immunohistochemistry staining of HMGA2 along with indicated markers in two GBM specimens. Please note high expression levels of these markers in highly-invasive (necrotic/pseudopalisading) regions. **E.** Kaplan-Meier survival plots showing correlations of glioma patients' survival with expression levels of *HMGA2* (probes A_23_P95930 and A_24_P85099) using the CGGA (Chinese Glioma Genome Atlas) dataset. Samples with *HMGA2* expression levels lying in top and bottom half are defined as $HMGA2^{High}$ and $HMGA2^{Low}$ groups respectively (n = 110 patients each). LV, lateral ventricle; IUE, in utero electroporation. Scale bars: (A) 500 μm, (B-D) 100 μm.

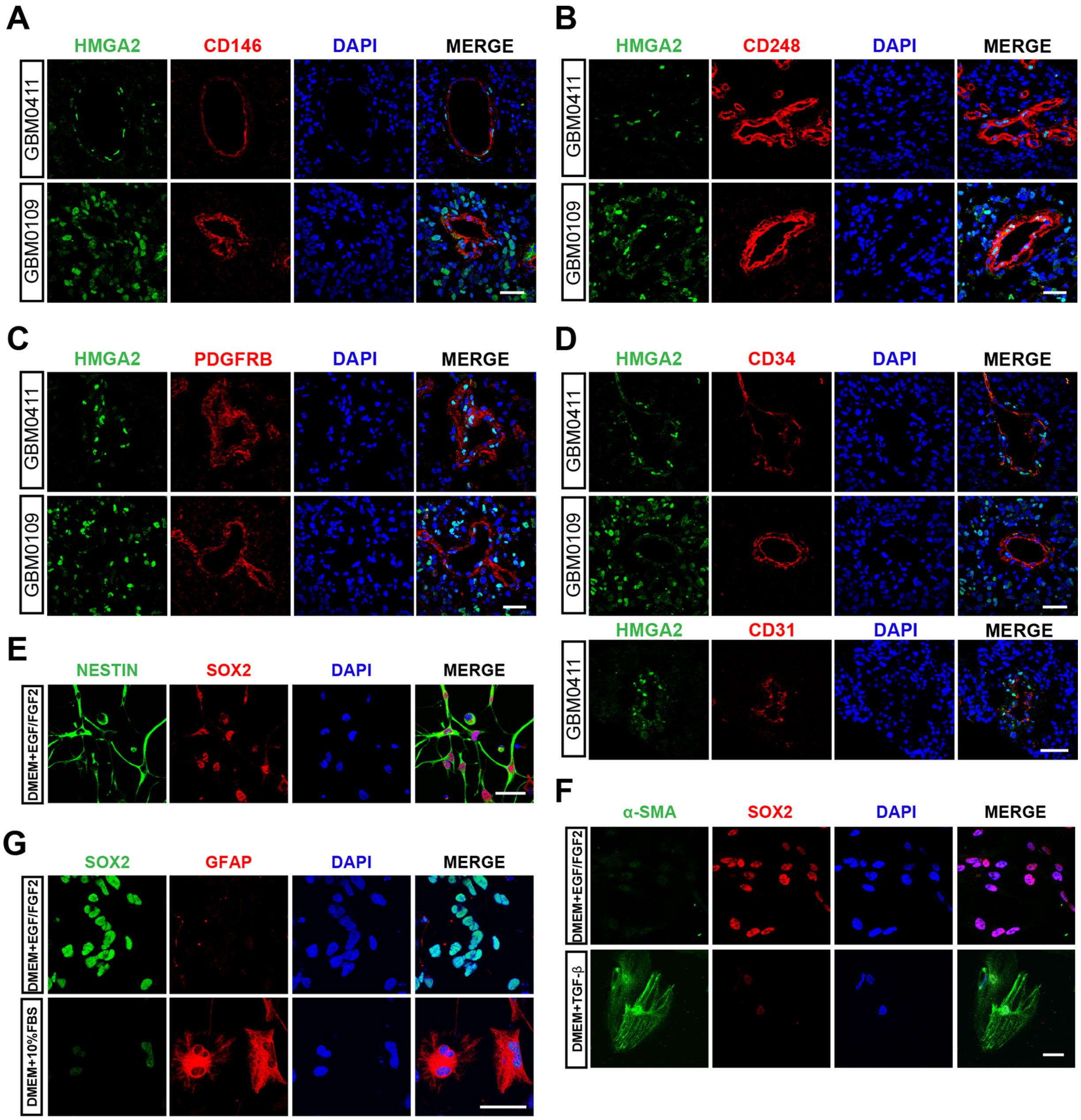

Supplementary Figure S2: HMGA2 labels GBM pericytes but not endothelial cells (ECs). A-D. Representative immunofluorescent images showing a large fraction of HMGA2 positive GBM cells co-express pericytes markers, CD146 (A), CD248 (B) and PDGFRB (C), but not EC markers CD34 and CD31 (D). **E.** Adherent cultured TPC1115 GICs supplemented with mitogens were double labeled with NESTIN and SOX2. **F.** Adherent cultured TPC1115 GICs were maintained in mitogenic conditions (top) or under TGF-β induced differentiation for 6 days (bottom) before double-labeling with SOX2 and α–SMA. **G.** Adherent cultured GICs were maintained in mitogenic conditions (top) or under 10% FBS induced differentiation for 5 days (bottom) before double-labeling with SOX2 and GFAP. Scale bars: 50 µm.

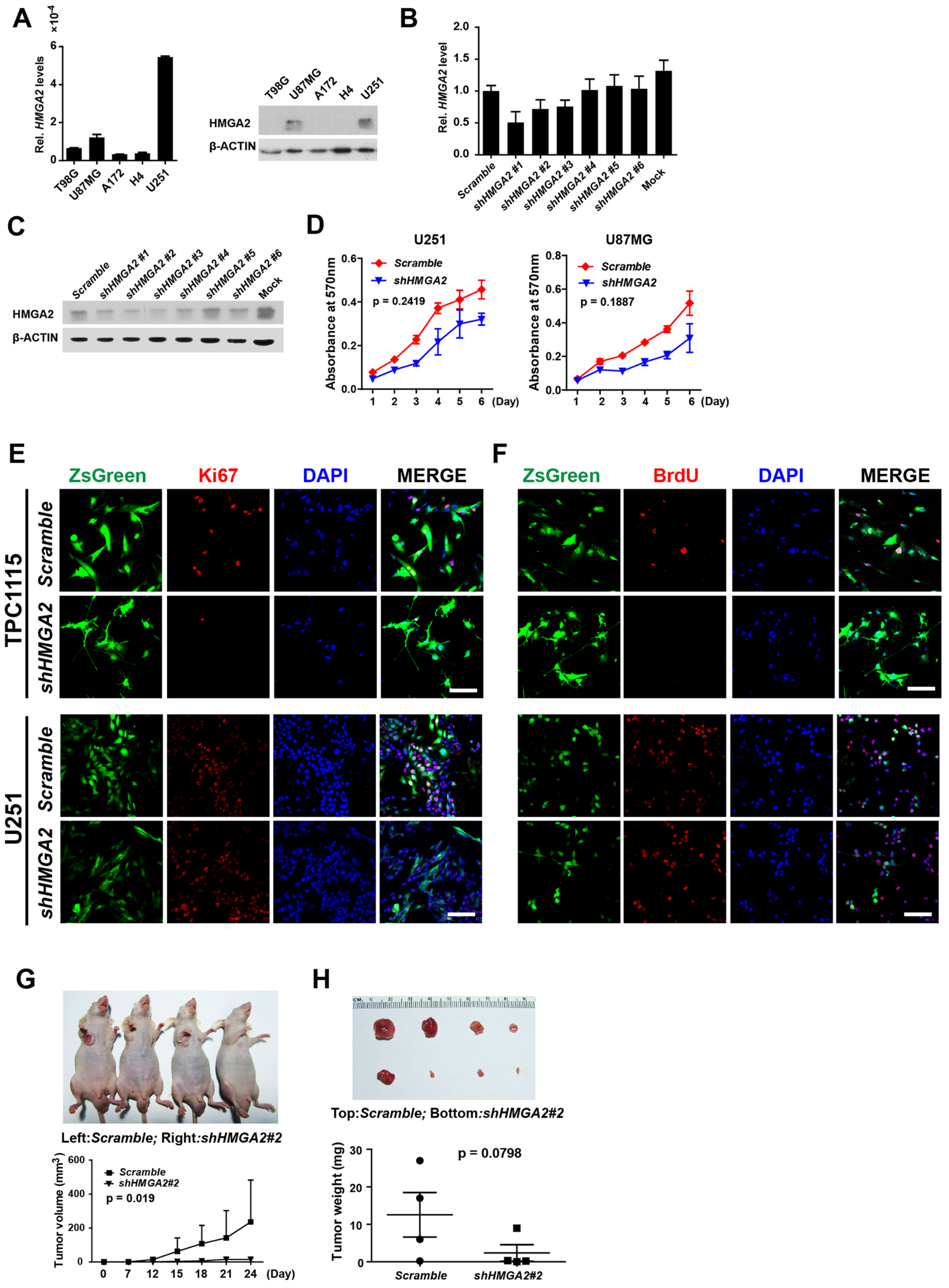

Supplementary Figure S3: HMGA2 knockdown impairs tumorigenicity of glioma cells. **A.** Expression of HMGA2 transcripts (left, relative to *β-actin*) and proteins (right) in glioma cell lines. **B-C.** Effects of shRNAs against *HMGA2* on its mRNA (B) and protein (C) in U251 glioma cells. **D.** MTT assays show effects of shHMGA2#2 on proliferations of U251 and U87MG glioma cells. **E-F.** Representative Immunofluorescent images showing Ki67 (E) and BrdU (F) staining of TPC1115 GICs and U251 glioma cells transduced with indicated lentiviruses that also express ZsGreen. BrdU were added into culture medium to a final concentration of 3 µg/mL three hours before fixation. **G.** Top, Images of nude mice three weeks after subcutaneous inoculation of 1×10^6 U251 glioma cells. Bottom, growth curves for xenografted U251 cells transduced with scramble- and shHMGA2-lentiviruses. **H.** Top, image of tumor mass resected from (G). Bottom, Scattered dot blot showing tumor weights distributions.

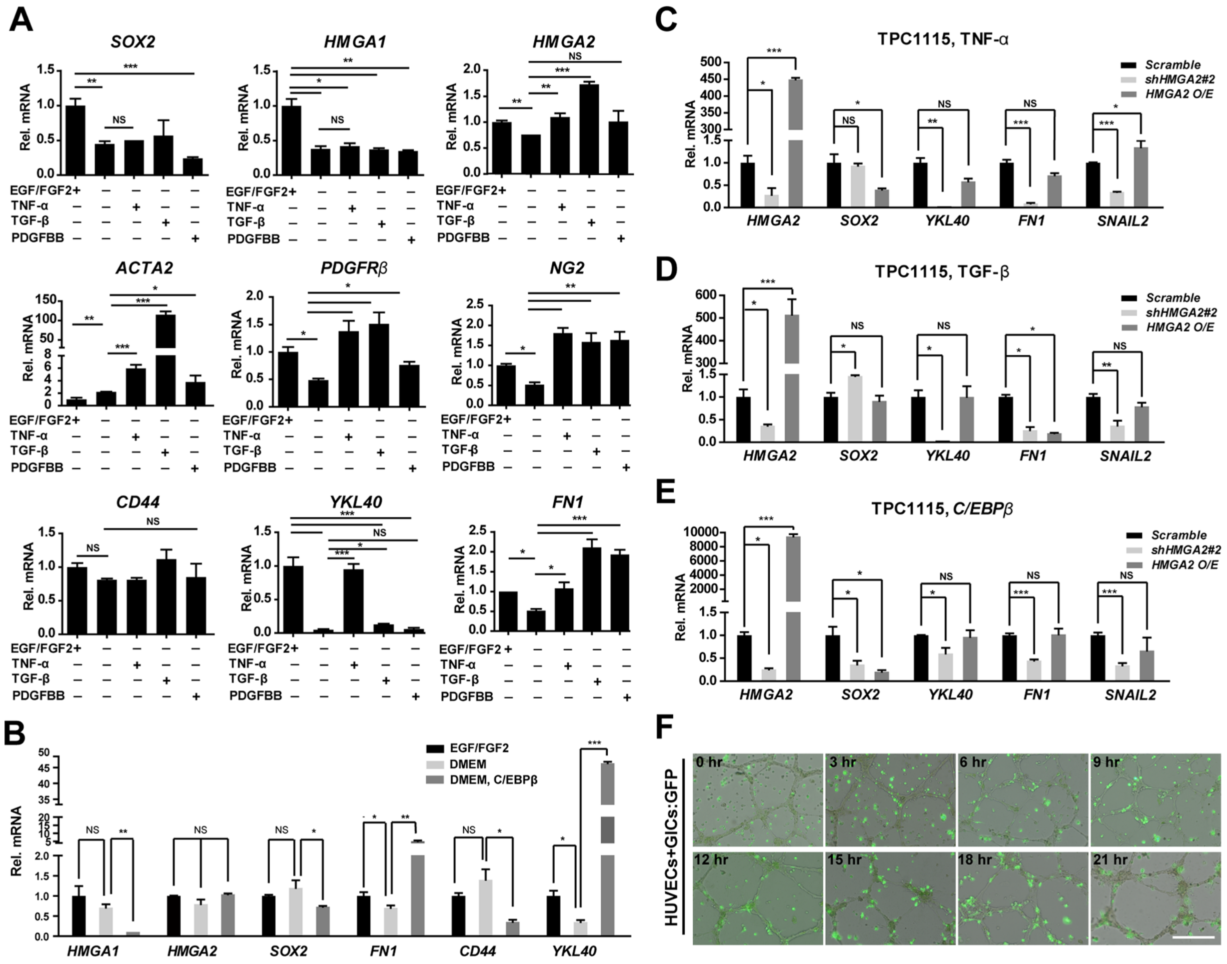

Supplementary Figure S4: HMGA2 is essential for mesenchymal and pericyte differentiation of GICs. A. Quantification and statistical analyses of expression levels of listed genes in TPC1115 GICs on indicated treatments. **B-E.** Quantification and statistical analyses of expression levels of listed genes in TPC1115 GICs on indicated treatments combined with HMGA2 knockdown or overexpression (O/E). **F.** HUVECs were plated onto matrigel to form endothelial complexes for 16-18 hours. Next, scramble-shRNA transduced TPC1115 GICs (ZsGreen+) were plated onto endothelial complexes. Representative images showing merged images at indicated time points. Scale bar: 300 μm.

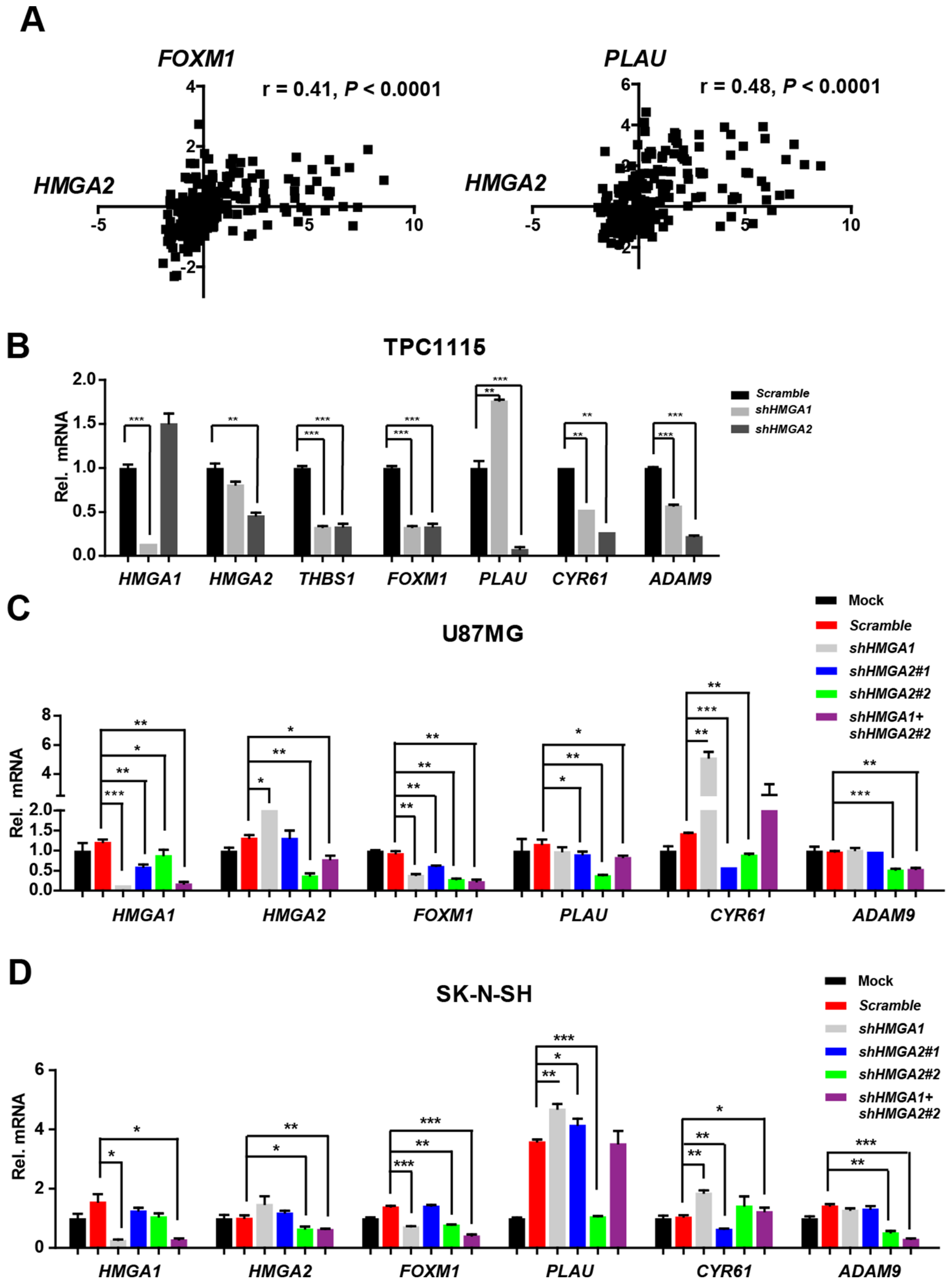

Supplementary Figure S5: Validation of candidate HMGA2 targets. A. Plots showing positive correlations of expression levels between *HMGA2* and *FOXM1* or *PLAU* in glioma samples using the CGGA dataset (n = 220). **B-D.** TPC1115 GICs (B), U87MG glioma cells (C) and SK-N-SH neuroblastoma cells (D) were transduced with indicated lentiviruses for 72 hours. Extracted RNAs were subjected to quantitative RT-PCR to measure expression levels of indicated transcripts.

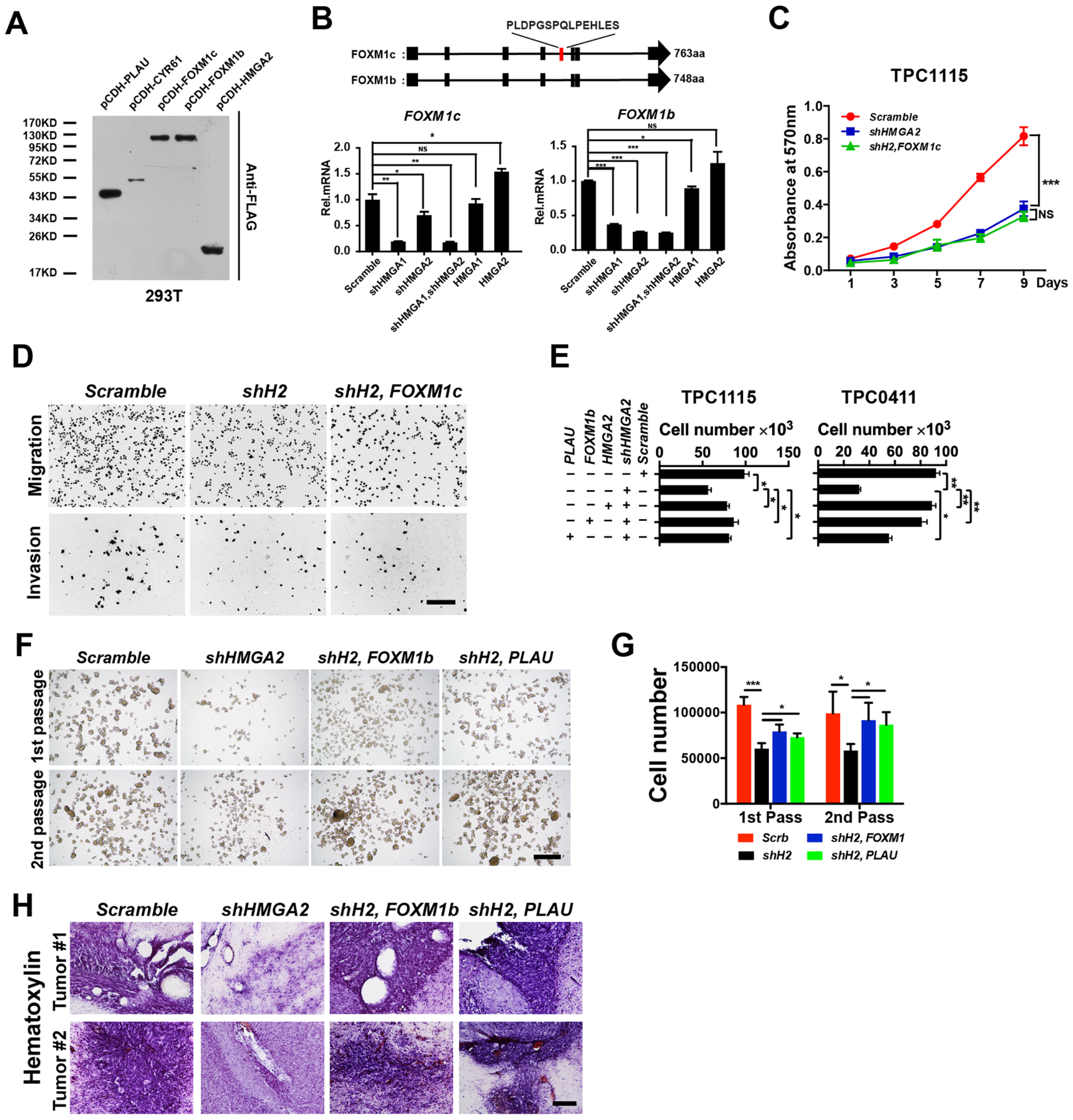

Supplementary Figure S6: Functional rescue of *HMGA2*-depleted GICs by FOXM1 and PLAU. A. Immunoblotting of protein extracts from 293T cells transfected with indicated plasmids expressing FLAG-tagged molecules. **B.** Quantification and statistical analyses of expression levels of two isoforms of *FOXM1* in TPC1115 GICs upon indicated treatments. **C.** MTT assay showing effects of *HMGA2* knockdown and FOXM1c rescuing on GIC propagation in adherent culture conditions. **D.** Representative images showing migratory and invasive TPC1115-derived cells transduced with indicated lentiviruses in transwell assays. Trans-welled cells were stained with DAPI for counting. **E.** TPC1115 and TPC0411 GICs cultured in neurosphere conditions for 7 days after transducing with indicated lentiviruses. Bar diagrams showing quantification of cell numbers of three independent experiments in Figure 6D. **F.** Representative images showing neurosphere cultures of TPC1115 GICs transduced with indicated lentriviruses for 7 days. Cells in the second passage were treated with lentiviruses expressing shHMGA2 for two consecutive passages. **G.** Quantifications of cell numbers in (F) (n = 3 experiments). **H.** The brains of Balb/c athymic nude mice were implanted with TPC1115 GICs expressing indicated lentiviruses. Eight weeks later, brains were dissected out and subjected to cryosectioning and hematoxylin stainings. shH2, shHMGA2. Scale bar: (D) 200 µm; (F) 500 µm; (H) 100 µm.

Supplementary Table S1A: List of genes downregulated following *HMGA1* and/or *HMGA2* depletion

**Cell cycle**	*MCM6, SKA3, SGOL2, CCNE2, CDCA8, BRCA2, MCM2, CLSPN, E2F8, BIRC5, CCNA1, GTSE1, CCND1, EXO1, PBK, CDC45, E2F1, SMC4, THBS1, FANCI, ASPM, FOXM1, DSN1, CIT*

Supplementary Table S1B: List of genes downregulated specifically following *HMGA2* depletion

**Cell migration and motility**	*PPAP2B, SMO, VCAM1, PARP1, DCC, MIXL1, PLAU, ROCK1, ITGA4, ADAM9*
**Regulation of apoptosis**	*SMO, NRAS, ATP7A, SOCS3, NGFR, CDK1, TERT, ROCK1, ADAM9, CDKN2C, P2RX7*
**Blood vessel development**	*CYR61, PPAP2B, SMO, ATP7A, PLAU, ITGA4*

* Genes listed in red were validated using qRT-PCR
